# All-inorganic perovskite photovoltaics for power conversion efficiency of 31%

**DOI:** 10.1038/s41598-023-42447-w

**Published:** 2023-09-14

**Authors:** Lipsa Rani Karna, Rohitash Upadhyay, Avijit Ghosh

**Affiliations:** Department of Physics, Central University of Jharkhand, Ranchi, 835222 India

**Keywords:** Materials science, Materials for devices

## Abstract

The lead-free perovskite halides emerge as the great alternative for highly efficient and environment friendly photovoltaics due to the inherent optoelectronic properties. In this paper, the numerical study of all-inorganic regular n–i–p structured perovskite photovoltaics using solar cells capacitance simulator (SCAPS-1D) has been performed. The optimised device structure using rGO provided best performance compared to the other hole transport layers (HTLs) like CuI, CuSCN, Cu_2_O, NiO, WSe_2_, MoO_3_ with CsSnI_3_ as an active material and TiO_2_ as electron transport layer (ETL). Furthermore, WS_2_ as an ETL compared to TiO_2_, Li-TiO_2_, ZnO, Al-ZnO, etc. provided the best performance with rGO as HTL and CsSnI_3_ as active material. Therefore, the optimized solar cell structure (FTO/WS_2_/CsSnI_3_/rGO/Pt) showed best photovoltaic performance with power conversion efficiency (PCE) of 31%, fill factor (FF) of 88.48%, open circuit voltage (V_OC_) of 1.15 V, and short circuit current density (J_SC_) of 30.47 mA/cm^2^, respectively. Consequently, the effect of variation of temperature, thickness, defect density, doping density of active layer and variation of illumination intensity on the photovoltaic performance of the optimised device are also analysed. Furthermore, this study is also focused on the analysis of photovoltaic parameters for the optimized structure using concept of ideality factor associated with the illumination intensity. Therefore, this analysis suggests a route for further development of all-inorganic, lead-free perovskite photovoltaics experimentally with improved photovoltaic performance.

## Introduction

The solar energy has great importance for the fulfilment of global energy demand in near future. In recent days single junction Si-solar cell contributes 90% of the photovoltaics (PV) market globally. The current power conversion efficiency (PCE) of crystalline Silicon (C-Si) solar cell is 27.6%^1^ approaching the theoretical limit to 33%^2^. Due to high manufacturing cost and installation of large area, the researchers are in search for alternatives to Si-solar cell. Therefore, recent development in this area have led to a new type of solar cell known as perovskite solar cell^3^. In this context, perovskite halides (chemical formula of ABX_3_, where A and B are metal cations and X is a halogen anion, respectively) have emerged as excellent candidate for photovoltaic (PV) energy conversion because of their low cost, ease of fabrication, flexibility, and versatility, etc. Apart from that, these have high absorption coefficient, excellent carrier mobility, long carrier diffusion length, low exciton binding energy, low trap state density, and tuneable bandgap, which enhance the PCE of device^4^. Here, the A-site usually includes monovalent cation like methylammonium (MA^+^), formamidinium (FA^+^), Cs^+^ or their mixture, meanwhile the halogen anion consists of Cl^−^, Br^−^, I^−^ or their mixture, and the B-site contains divalent cation generally Pb^2+^, Sn^2+^, etc.^5^. The PCE of perovskite solar cells (PSCs) based on lead (Pb) has been dramatically improved from the initial value of 3.8%^6^ to 25.8% till date^7^. But, due to lead toxicity, the recent research is focused on Pb^2+^-free perovskites like Sn^2+^, Bi^2+^, Sb^2+^, Ge^2+^, etc. based PV materials^5^. Recently, the highest PCE obtained by a Sn^2+^-based one is 14.81% having FASnI_3_ as an absorber material^8^. Although, the PCE is lower than that of lead based one, but more research is going on to increase its performance by replacing FA^+^ from A-site using inorganic cations like Cs^+^, Rb^+^, etc. to reduce the recombination effect for trap states of the absorber layer. It is reported that Sn^2+^ based perovskite i.e., CsSnI_3_ (both the β and γ phases) is an excellent candidate^9,10^ for photovoltaics due to their suitable band gap of 1.3 eV (close to the ideal value 1.34 eV) and high thermal stability up to 450 °C^11^. Other than the above, due to the extraordinary optical and electrical properties of carbon-based materials such as graphene, graphene oxide (GO), reduced graphene oxide (rGO), it can be used as an ETL, HTL and additive to the active layer for the PSCs. Higher PCE of 15.2% was achieved using perovskite along with GO as an active layer^12^. In several studies, GO is used with HTL layer (PEDOT: GO) to enhance the ambient stability^13^. Especially, rGO have tuneable electronic properties i.e., variable work function from 4.4 to 4.9 eV, for which it behaves as a good HTL. Also due to the low oxygen concentration it shows better thermal stability while using as an additive in PSCs^14^. The rGO based on graphene scaffold as interface layer between the ETL and active layer got achieved a PCE up to 17.2%^15^. It is found that rGO-HBS (rGO-4-hydrazino benzenesulfonic acid) as HTL in an inverted planar PSCs exhibits a PCE of 16.4%^16^. As the rGO has low oxygen content, which may lower the oxidation state of Sn^2+^ to Sn^4+^ in the interface of rGO-CsSnI_3_, whereas it enhances the stability of the device. Also, the increased hole extraction behaviour of rGO and presence of fewer trap states in the interface of HTL-perovskite layer increases PCE value of the device. Due to the excellent optoelectronic behaviour like maximum absorption and high carrier mobility in the rGO molecule, we consider it as a HTL in our model to enhance the efficiency of the device. Furthermore, among different transition metal dichalcogenides (TMDCs), tungsten disulfide (WS_2_) is implemented as an ETL due to its excellent behaviour towards conductivity (~ 10^–3^ Ω^−1^ cm^−1^) and carrier mobility^17^. Chalcogenide like WS_2_ is an

earth-abundant, direct band gap semiconducting material with non-toxic and adhesive properties and for better carrier conduction behaviour, it is used as an ETL in lead-free PSC having Sb_2_Se_3_ as an active layer^18^. Furthermore, due to its layer translational properties and lattice matching with CsPbI_3_ active layer, it is used as a lubricant in between the substrate and active layer in an inorganic CsPbI_3_ based solar cell to bring down the tensile strain developed between the interface of ETL and perovskite layer^19^. Its tuneable band gap between 1.3 and 2.2 eV helps for easy conduction of electron in the solar cell^20^. It is also reported that using 2D WS_2_ nanosheet as an ETL in PSC achieved a PCE of 18.21% due to its high interfacial carrier extraction properties in PSCs^21^. It can be deposited by solution processed low temperature technique or RF sputtering method^22^. Thus, it may be considered as a good candidate for practical application as ETL in the fabrication of PSCs.

There are several attempts both theoretically and experimentally to enhance the PV performances in PSCs. Under optimized conditions, the FTO/SnO_2_ (25 nm)/CsPbI_3_ (700 nm)/Cu_2_O (170 nm)/Au all-inorganic PSC provided the PCE of 21.31%^23^. The HTL free PSC with gradient doping structure and gradient energy band structure provided the PCE of 21.15% for FTO/TiO_2_/interface defect layer (IDL)/CsPbI_3_ (1000 nm)/carbon based device structure^24^. Furthermore, the obtained conversion efficiency of 10.11% from the Pb-free, the optimised device structure on FTO/TiO_2_ (50 nm)/RbGeI_3_ (500 nm)/NiO (100 nm)/Ag is also reported^25^. The device with FTO/TiO_2_ (10 nm)/CsGeI_3_ (800 nm)/CuI (30 nm)/C structure exhibited an optimized PCE of 10.8% with a QE of 95% in the visible range^26^. The PCE of ~ 19.08% was achieved by Pb-free optimised FTO/TiO_2_ (10 nm)/FASnI_3_ (~ 2000 nm)/Spiro-OMeTAD (50 nm)/Au with the effect of variation in the concentration of defect density, doping of absorber, affinity of ETL, HTL and capture cross section concentration^27^. The MAGeI_3_-based solar cell device architecture [FTO (500 nm)/ZnSe (50 nm)/MAGeI_3_ (500 nm)/Cu_2_O (350 nm)] provided that an excellent open circuit voltage (V_oc_) of 1.37 V and J_SC_ value of 14.22 mA cm^−2^ with a PCE of 17.61%^28^. Furthermore, using ZnO (ETL) and Cu_2_FeSnS_4_ (CFTS) (HTL) as charge transport layers, the combination with ITO (500 nm)/ZnO (50 nm)/CsPbI_3_ (800 nm)/CFTS (100 nm)/Au structure with a V_OC_ of 0.57 V and J_SC_ of 25.02 mA cm^−2^ provided the PCE of 7.38% only^29^. Meanwhile, using the same charge transport layers, the perovskite absorber CsSnI_3_ (800 nm) exhibited that the PCE of 7.97%^29^.

The optimised simulated structure based on ITO (500 nm)/PCBM (50 nm)/CsSnI_3_ (1000 nm)/CFTS (200 nm)/Se provided a PCE of 24.73%^30^. Also, by considering inorganic TiO_2_ as ETL and organic Spiro-OMeTAD as HTL a PCE of 28.76% was obtained from a device structure of CuS (100 nm)/B-γ-CsSnI_3_ (600 nm)/TiO_2_/(50 nm) ITO (50 nm) after minimization of the recombination of charge carrier at the HTL/perovskite interface^31^. Another attempt to obtain PCE of 26.4% has been optimised through FTO/TiO_2_ (40 nm)/CsSnI_3_ (800–1000 nm)/P3HT (200 nm)/Au device structure with a direct bandgap of 1.3 eV in CsSnI_3_ absorber layer^32^. Very recent study for the optimized structure on FTO/n-TiO_2_ (50 nm)/CsSnI_3_ (1250 nm, E_g_ ~ 1.35 eV)/p-NiO (50 nm)/Au provided the PCE of 31.09%^33^. It is also reported that perovskite layer thickness is inappropriate using thinner (< 200 nm) or thicker (> 700 nm) film in the device. If the thin layer used, the low photocurrent results due to less absorption, but carrier extraction is high. Meanwhile, for the thick perovskite layer, although more carriers are generated in the device due to an increase in absorption, lower collection efficiency is because of recombination which affects the Voc^34^. Therefore, to achieve the excellent PV performance, it is required to improve the band alignment with absorber layer into ETL and HTL by optimizing the several input parameters like total defect density, band gap of active layer, acceptor level density, donor level density, and suitable film thickness of each layer, etc.

Other than the above, the Pb-based PSCs for PCE of 18.8%, 19%, 20.1%, 20.4%, and 21% on FTO/c-TiO_2_/PCBA/CsPb_0.95_Ge_0.05_I_3_/spiro-OMeTAD/Au^35^, FTO/TiO_2_/CsPbI_3_/spiro-OMeTAD/Ag^36^, FTO/TiO_2_/SRL/CsPbI_3_/spiro-OMeTAD/Au^37^, FTO/TiO_2_/CsPbI_3_/OAI/spiro-OMeTAD/Au^38^, and FTO/TiO_2_/CsPbI_3_/PTAI/spiro-OMeTAD/Au^39^ structures have been demonstrated practically. Furthermore, the Pb-free PCE of 7.2%, and 10.7% was also achieved in ITO/PEDOT:PSS/FA_0.75_MA_0.25_SnI_3_/Bathocuproine (BCP)/C_60_/Ag^40^ and ITO/PEDOT:PSS/FASnI_3_/C_60_/BCP/Ag^41^ structures, respectively. A few attentions have been also paid to Pb-free all-inorganic PSCs based on CsSnI_3_ thin films. Chen et al. demonstrated that PCE of 7.11% for FTO/PCBM/CsSn_0.5_Ge_0.5_I_3_/Native Oxide/Spiro-OMeTAD/Au device structure^42^.

In view of the above, to achieve a remarkable PCE value for lead-free PSC, we have proposed a model of n–i–p structure simulated by SCAPS-1D simulation software. The variation of HTL and ETL is performed taking CsSnI_3_ as an absorber layer. Where 2D layered structured materials like rGO and WS_2_ are used as HTL and ETL, respectively. Therefore, the novelty of the work is to study the photovoltaic performance of optimised structure in comparison with the lead based one. Furthermore, a theoretical study of different PV parameters is performed by varying the absorber layer thickness, defect density, carrier doping density and probe temperature of the device. In addition to that, the obtained electrical and optical parameters from the above study can provide us an overall knowledge about ideality factor in association with recombination phenomena occurring inside the device, which gives deep understanding for experimental approach in device fabrication.

## Device structure and simulation parameters

**Figure 1:**
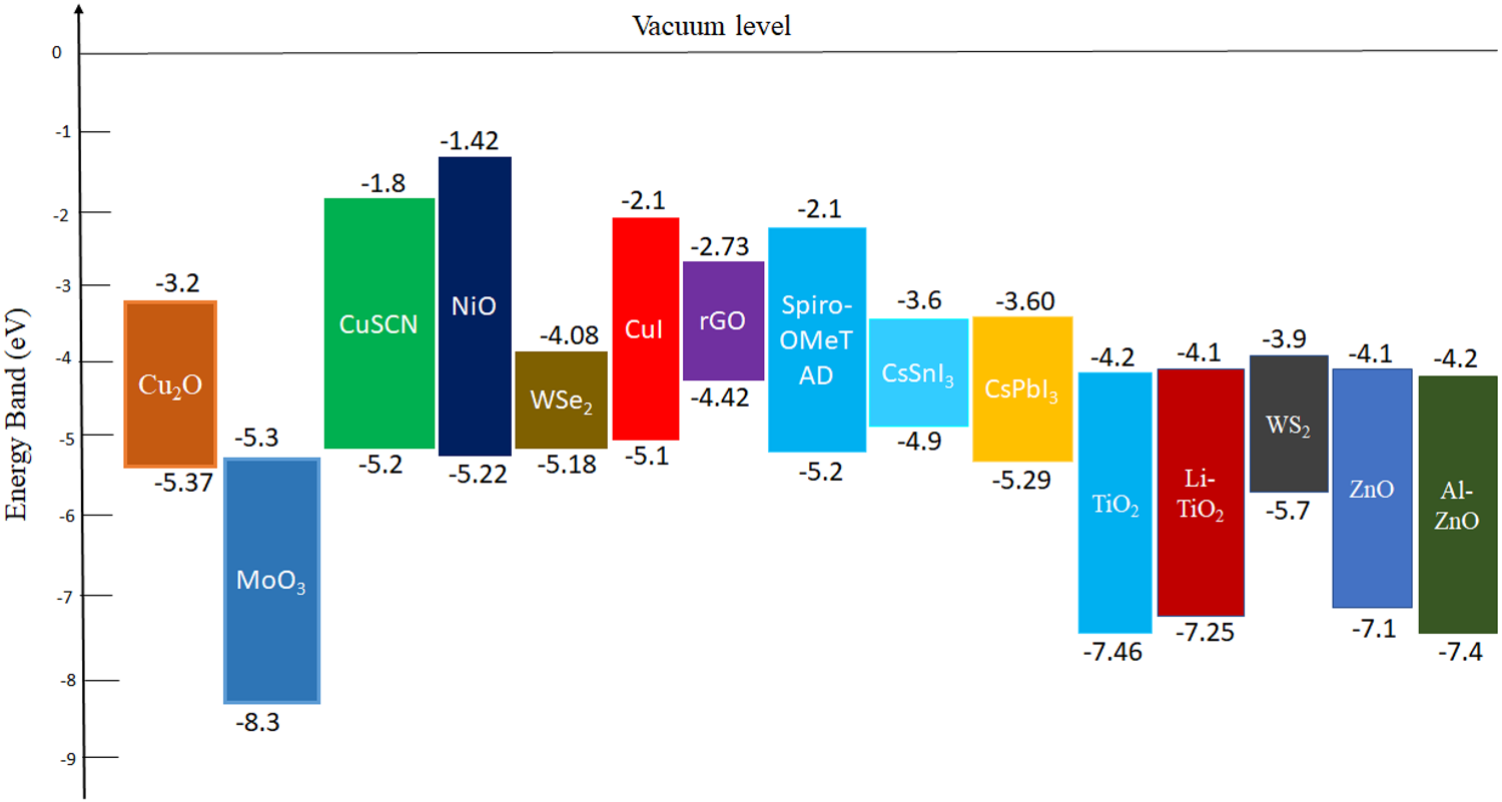
Energy levels of different HTLs, ETLs, CsPbI_3_ and CsSnI_3_ (active layers).

**Figure 2:**
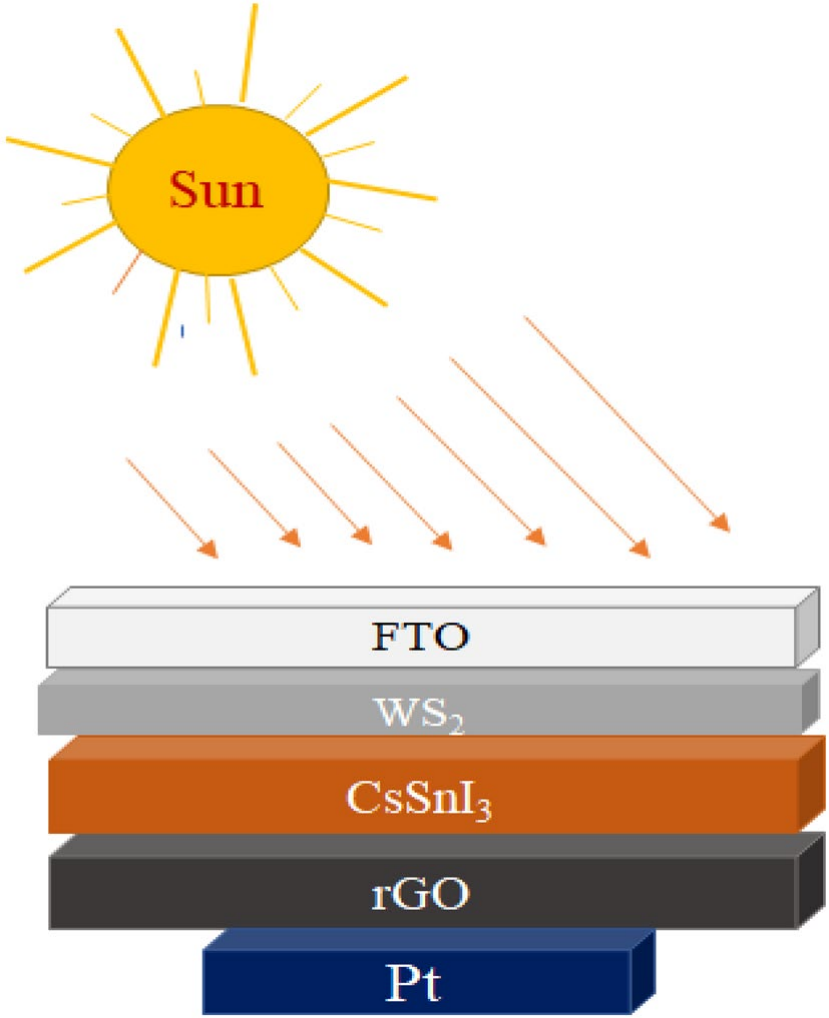
Optimised device structure of lead-free all-inorganic PSC.

To evaluate or estimate the photovoltaic performance of different PSCs, device structures are simulated by various simulation software like SCAPS-1D, PC-1D, AMPS-1D, wxAMPS, COMSOL, Silvaco, etc.^43^. Among which SCAPS-1D version-3.3.09 is used in this study for simulating and modelling the PSC having CsSnI_3_ as the active layer. This version is developed by the Department of Electronics and Information Systems (ELIS), University of Gent, Belgium^44,45^. The simulation is based on SCAPS-1D for multijunction (tandem) as well as single junction solar cell using three semiconductor equations. Equation (1) corresponding to Poisson equation^46^ provides the relation between carrier concentrations and electrostatic potential. Equations (2) and (3) refer to the continuity equations for electrons and holes, respectively^43^ for the relation between charge carrier generation and recombination mechanism in semiconductor. All the equations are shown below:

1 $$\frac{d}{dx}\left(-\varepsilon \left(x\right)\frac{d\psi }{dx}\right)=q\left[p\left(x\right)-n\left(x\right)+{N}_{D}^{+}\left(x\right)-{N}_{A}^{-}\left(x\right)+{p}_{t}\left(x\right)+{n}_{t}\left(x\right)\right]$$

2 $$\frac{d{J}_{n}}{dx}=G-R$$

3 $$\frac{d{J}_{p}}{dx}=G-R$$

where ɛ and ψ represent the permittivity and electric potential, q is charge of electron, n(x) and p(x) refer to the concentration of electron and hole, N_D_^+^ and N_A_^−^ represent the donor and acceptor doping density, n_t_(x) and p_t_(x) show the defect density, R refers to recombination rate of electron and hole, G is the generation rate of excitons, J_n_ and J_p_ are the current density of electron and hole, respectively. The charge transport mechanism can be understood using drift–diffusion model to be determined by the following Eqs. (4) and (5)^45,47^:

4 $${J}_{n }= q{D}_{n}\frac{dn}{dx}+q{\mu }_{n}n\frac{d\psi }{dx}$$

5 $${J}_{p}= {qD}_{p}\frac{dp}{dx}+ {q\mu }_{p}p\frac{d\psi }{dx}$$

where D_n_ and D_p_ are the diffusion coefficient for electron and hole, µ_n_ and µ_p_ are electron and hole mobility, respectively. The SCAPS-1D is performed using the following Eq. (6) for the calculation of absorption coefficient^4^:

6 $$\alpha \left(\lambda \right)=\left(A+\frac{B}{h\nu }\right)\sqrt{h\nu -{E}_{g}}$$

where α is the absorption coefficient (function of wavelength), A and B are the constants which is generally taken as 10^+5^ and 10^–12^, respectively, h is the Planck’s constant, ν is the incident photon frequency and E_g_ denotes the band gap of absorbing layer. The various models having CsSnI_3_ as an active layer has been simulated using several HTLs and ETLs in the present study. The HTLs (p-type) extract holes from the active layer to electrode for functioning the PSC. An efficient HTL must have high charge carrier mobility and high work function for the easy transportation of carriers^48^. Here, we have compared photovoltaic performance of the device for different inorganic HTLs like Cu_2_O, NiO, CuSCN, WSe_2_, CuI, MoO_3_, rGO and for organic HTL (Spiro-OMeTAD) whereas TiO_2_ used as the ETL. Due to the high electron extraction capacity of TiO_2_ (n-type) from the perovskite absorber layer with an appropriate band-alignment for blocking the holes, it can reduce the hysteresis loss to increase the PCE and stability of the PSCs^46,49^. Furthermore, a comparison is drawn between the device for different ETLs like Li-TiO_2_, WS_2_, ZnO, Al-ZnO meanwhile taking rGO as HTL. For ETL, generally we used metal oxides having semiconducting properties like ZnO, TiO_2_, SnO_2_ and their doped ones. But evolution of surface defects and oxygen vacancies by these materials can be minimised by replacing these with TMDCs like WS_2_ as ETLs^21^. Another comparison is done between lead-free (CsSnI_3_) and lead based (CsPbI_3_) active material with structure FTO/WS_2_/Perovskite/rGO. All the physical parameters are listed in Tables 1, 2, and 3 for the structural simulation of PSC using SCAPS-1D. All the basic simulation process is performed under AM 1.5G (1000 W m^−2^) illumination intensity and at room temperature (RT). Furthermore, the intensity of illumination and probe temperature are varied to evaluate its effect on the photovoltaic parameters like V_OC_, PCE, J_SC_ and FF of the PSC. Also, the impact of thickness variation, doping density, and defect density of absorber layer on the given PSC parameters have been analysed. In the SCAPS-1D action panel, thermal velocity of electron and hole are taken to be 1.0 × 10^7^ cm s^−1^. The defect type is selected to be neutral, meanwhile, the band-to-band recombination data has been ignored in this simulation. The single energetic distribution is taken with characteristic energy of 0.1 eV for all the layers, whereas series resistance (R_S_) and shunt resistance (R_Sh_) are ignored in the initial calculations. In this simulation, the optical reflection from each layer and interfaces are not included, and the work function of the front contact (FTO) is taken as 4.4 eV with 100% transmission as a default value. Figure 1 displays the energy level diagrams of different HTLs, ETLs, CsSnI_3_ and CsPbI_3_ layer. Furthermore, Fig. 2 gives the optimised device structure representation of lead-free all-inorganic PSC.

**Table 1:** Simulation parameters for different layers of PSC.

Parameters	FTO^50^	WS_2_^51^	CsSnI_3_^50^	rGO (this work)
Thickness (nm)	400	100	400	350
Band gap (E_g_) (eV)	3.5	1.8	1.3	1.69^52^
Electron affinity (χ) (eV)	4	3.95	3.9	3.56^53^
Dielectric permittivity	9	13.6	18	13.3^54^
Conduction band DOS (N_c_) (cm ^−3^)	2.02 × 10^18^	10^18^	2.2 × 10^17^	10^18^
Valence band DOS (N_v_) (cm ^−3^)	1.8 × 10^19^	10^18^	1.8 × 10^19^	1.8 × 10^19^
Thermal velocity of electron (V_th,e_) (cm s^−1^)	10^7^	10^7^	10^7^	10^7^
Thermal velocity of hole (V_th,h_) (cm s^−1^)	10^7^	10^7^	10^7^	10^7^
Electron mobility (µ_e_) (cm^2^ Vs^−1^)	2 × 10^1^	50	2 × 10^–1^	2.6 × 10^1^^55^
Hole mobility (µ_h_) (cm^2^ Vs^−1^)	1 × 10^–1^	50	2 × 10^–1^	1.23 × 10^2^^56^
Donor density (N_D_) (cm^−3^)	2 × 10^19^	10^18^	0	0
Acceptor density (N_A_) (cm^−3^)	0	0	2 × 10^18^	10^22^^55^
Total defect density (N_t_) (cm^−3^)	10^15^	10^14^	10^14^	10^14^

**Table 2:** Simulation parameters of different HTLs and CsPbI_3_ (active) layer.

Parameters	CuSCN^57^	NiO^58^	CuI^59^	Cu_2_O^60^	MoO_3_^61^	WSe_2_^62^	Spiro-OMeTAD^4^	CsPbI_3_^63^
Thickness (nm)	350	350	350	350	350	350	350	400
E_g_ (eV)	3.4	3.80	3.1	2.17	3	1.55	2.9	1.69
χ (eV)	1.9	1.46	2.1	3.2	2.5	4.03	2.2	3.95
ɛ_r_	10	11.75	6.5	7.1	12.5	13.8	3.0	6
N_c_ (cm^−3^)	1.7 × 10^19^	2.2 × 10^20^	2.2 × 10^19^	2.5 × 10^18^	2.2 × 10^18^	8.3 × 10^18^	2.2 × 10^18^	1.1 × 10^20^
N_v_ (cm^−3^)	2.5 × 10^21^	1.8 × 10^19^	1.8 × 10^19^	1.8 × 10^19^	1.8 × 10^19^	1.4 × 10^19^	2.2 × 10^18^	8.2 × 10^20^
V_th,e_ (cm s^−1^)	10^7^	10^7^	10^7^	10^7^	10^7^	10^7^	10^7^	10^7^
V_th,h_ (cm s^−1^)	10^7^	10^7^	10^7^	10^7^	10^7^	10^7^	10^7^	10^7^
µ_e_ (cm^2^ Vs^−1^)	10^–4^	2.8	100	2 × 10^2^	25	30	10^–4^	25
µ_h_ (cm^2^ Vs^−1^)	10^–1^	2.8	43.9	8 × 10^2^	100	30	10^–4^	25
N_D_ (cm^−3^)	0	0	0	0	0	0	0	0
N_A_ (cm^−3^)	10^18^	10^18^	10^18^	9 × 10^21^	10^18^	10^16^	1.3 × 10^18^	10^15^
N_t_ (cm^−3^)	10^14^	10^15^	10^14^	10^14^	10^15^	10^14^	10^15^	2 × 10^14^

**Table 3:** Simulation parameters of different ETLs.

Parameters	ZnO^64^	Al-ZnO^64^	TiO_2_^65^	Li-TiO_2_^65^
Thickness (nm)	100	100	100	100
Band gap (E_g_) (eV)	3.28	3.1	3.15	3.15
Electron affinity (χ) (eV)	4	4	4	4
Dielectric permittivity	9	9	10	13.6
Conduction band DOS (N_c_) (cm^−3^)	2 × 10^18^	2 × 10^18^	2.5 × 10^19^	3 × 10^20^
Valence band DOS (N_v_) (cm^−3^)	1.8 × 10^19^	1.8 × 10^19^	3 × 10^19^	2 × 10^20^
Thermal velocity of electron (V_th,e_) (cm s^−1^)	10^7^	10^7^	10^7^	10^7^
Thermal velocity of hole (V_th,h_) (cm s^−1^)	10^7^	10^7^	10^7^	10^7^
Electron mobility (µ_e_) (cm^2^ Vs^−1^)	43	13.84	2 × 10^–4^	30
Hole mobility (µ_h_) (cm^2^ Vs^−1^)	25	25	3 × 10^–6^	0.005
Donor density (N_D_) (cm^−3^)	2.9 × 10^15^	1.02 × 10^19^	2 × 10^17^	8 × 10^17^
Acceptor density (N_A_) (cm^−3^)	0	0	0	0
Total defect density (N_t_) (cm^−3^)	10^15^	10^15^	10^14^	10^14^

## Results and discussion

**Figure 3:**
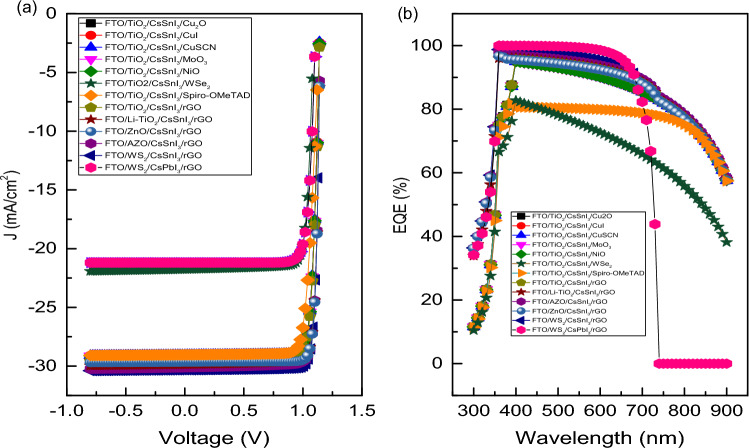
(**a**) J–V curves and (**b**) variation of external quantum efficiency curves for CsSnI_3_ and CsPbI_3_ based devices having different HTLs and ETLs.

**Figure 4:**
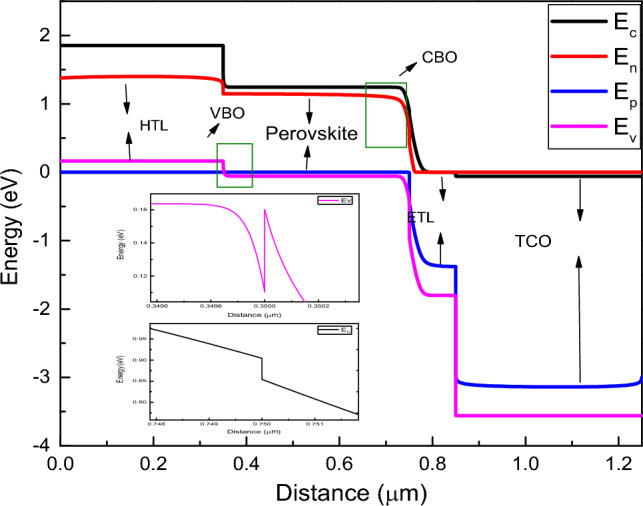
Energy band diagram for FTO/WS_2_/CsSnI_3_/rGO based device and representation of spike at perovskite/HTL and cliff at perovskite/ETL interface (inset).

Figure 3a,b exhibit the variation of current density with voltage (J–V) and the variation of external quantum efficiency with wavelength of incident light for different device structures. Also, Figure S1a shows the J–V curves and Figure S1b shows the variation of external quantum efficiency with wavelength for CsSnI_3_ and CsPbI_3_ based devices with rGO as HTL and WS_2_ as ETL**.** The external quantum efficiency (EQE) of any PSC is the spectral response or the current obtained by photon absorption from the incident solar spectrum^66^. The EQE spectra is related to short circuit current (J_SC_) of the device obeyed the following Eq. (7)^67^.

7 $${J}_{SC}=q\int F\left(\lambda \right).EQE\left(\lambda \right)d\lambda$$

where F(λ) represents the photon flux as a function of wavelength and q is the charge of free carriers. The computed result or PV parameters for different device structures are tabulated in Table 4. Best cell performance obtained from the above is for device structure FTO/WS_2_/CsSnI_3_/rGO with PCE = 30.84%, FF = 87.54%, J_SC_ = 30.31 mA cm^−2^ and V_OC_ = 1.162 V, which is taken as ideal structure for further analysis. Most importantly, using rGO as a HTL for both the CsSnI_3_ and CsPbI_3_ based devices, the EQE increases for wavelength ranging from 300 to 360 nm rapidly then it is almost constant up to 650 nm after which it decreases up to 900 nm. This trend shows the high photon absorption in visible region (400–700 nm) which is essential for better performance of PSCs. Figure 4 manifests the energy band diagram for simulated CsSnI_3_ based device using rGO as HTL and WS_2_ as ETL. It is observed that the band gap ranging from 1.3 to 2.15 eV for lead free PSCs gives better photovoltaic result, which is satisfied by our simulated device where the E_g_ for CsSnI_3_ (active layer) is taken as 1.3 eV and achieved a better PV performance. It is observed that increase in bandgap of active layer decreases the I_SC_ value due to decrease in absorption of photons whereas V_OC_ increases because of easy segregation of charge carriers inside the active layer^58^. Furthermore, to minimise the charge recombination and simultaneously to maximize carrier extraction from interfaces of different layers inside the device structure, the bandgap matching between HTL, ETL and absorber layer is very much essential. To achieve the above, conduction band offset (CBO) between ETL and absorber layer as well as valence band offset (VBO) between HTL and absorber come into consideration^68^. The above facts can be explained using the following Eqs. (8) and (9):

8 $$CBO=\left({\chi }_{Abs}-{\chi }_{ETL}\right)$$

9 $$VBO=\left({\chi }_{HTL}-{\chi }_{Abs}+{{E}_{g}}_{HTL}-{{E}_{g}}_{Abs}\right)$$

where χ_Abs_, χ_HTL_ and χ_ETL_ define the electron affinity of absorber layer, HTL and ETL, respectively. E_gHTL_ and E_gAbs_ denote the bandgap of HTL and absorber layer, respectively. The value of CBO and VBO depend on barrier height at interfaces formed by the photo generated carriers, which simply depends on the electron affinity of ETL, HTL and absorber layer as shown in the above-mentioned Eqs. (8) and (9). For better performance of PSC, the VBO and CBO should be a small positive value, as high value of VBO create hinderance for the conduction of hole from absorber to HTL and negative value of VBO enhance the carrier recombination^69^. Figure 4 shows the energy band diagram for FTO/WS_2_/CsSnI_3_/rGO based device and a spike at perovskite/HTL and cliff at perovskite/ETL interface (inset). Here, the E_c_, E_v_, E_n_, E_p_ represent the conduction band energy level, valence band energy level, Fermi level in n-type material and Fermi level in p-type material, respectively. From the Fig. 4, we find a cliff at the WS_2_ and CsSnI_3_ interface. This provides a negative value of CBO i.e., − 0.05 eV. This negative value of CBO allows the easy conduction of electrons from absorber to front contact through ETL. Moreover, we find a spike at the rGO and CsSnI_3_ interface, providing a positive value of VBO i.e., + 0.05 eV. However, this hinders the flow of holes from HTL to back electrode but this small VBO, may not impact the overall performance of the device.

**Table 4:** Photovoltaic performance of different device structures.

Device structure	HTL	ETL	FF (%)	V_OC_ (V)	J_SC_ (mA cm^−2^)	PCE (%)
FTO/TiO_2_/CsSnI_3_/Cu_2_O	Cu_2_O	TiO_2_	85.77	1.14	29.19	28.70
FTO/TiO_2_/CsSnI_3_/CuI	CuI	TiO_2_	85.79	1.14	29.05	28.55
FTO/TiO_2_/CsSnI_3_/CuSCN	CuSCN	TiO_2_	85.79	1.14	29.03	28.53
FTO/TiO_2_/CsSnI_3_/MoO_3_	MoO_3_	TiO_2_	85.79	1.14	29.07	28.59
FTO/TiO_2_/CsSnI_3_/NiO	NiO	TiO_2_	85.79	1.14	29.06	28.57
FTO/TiO_2_/CsSnI_3_/WSe_2_	WSe_2_	TiO_2_	84.39	1.09	21.76	20.18
FTO/TiO_2_/CsSnI_3_/Spiro-OMeTAD	Spiro-OMeTAD	TiO_2_	81.74	1.14	29.03	27.22
FTO/TiO_2_/CsSnI_3_/rGO	rGO	TiO_2_	85.75	1.14	29.49	29.00
FTO/Li-TiO_2_/CsSnI_3_/rGO	rGO	Li-TiO_2_	87.88	1.15	29.86	30.18
FTO/ZnO/CsSnI_3_/rGO	rGO	ZnO	88.87	1.14	29.65	30.30
FTO/Al-ZnO/CsSnI_3_/rGO	rGO	Al-ZnO	87.76	1.14	30.11	30.36
FTO/WS_2_/CsSnI_3_/rGO	rGO	WS_2_	87.54	1.16	30.31	30.84
FTO/WS_2_/CsPbI_3_/rGO	rGO	WS_2_	84.28	1.11	21.19	19.85

**Figure 5:**
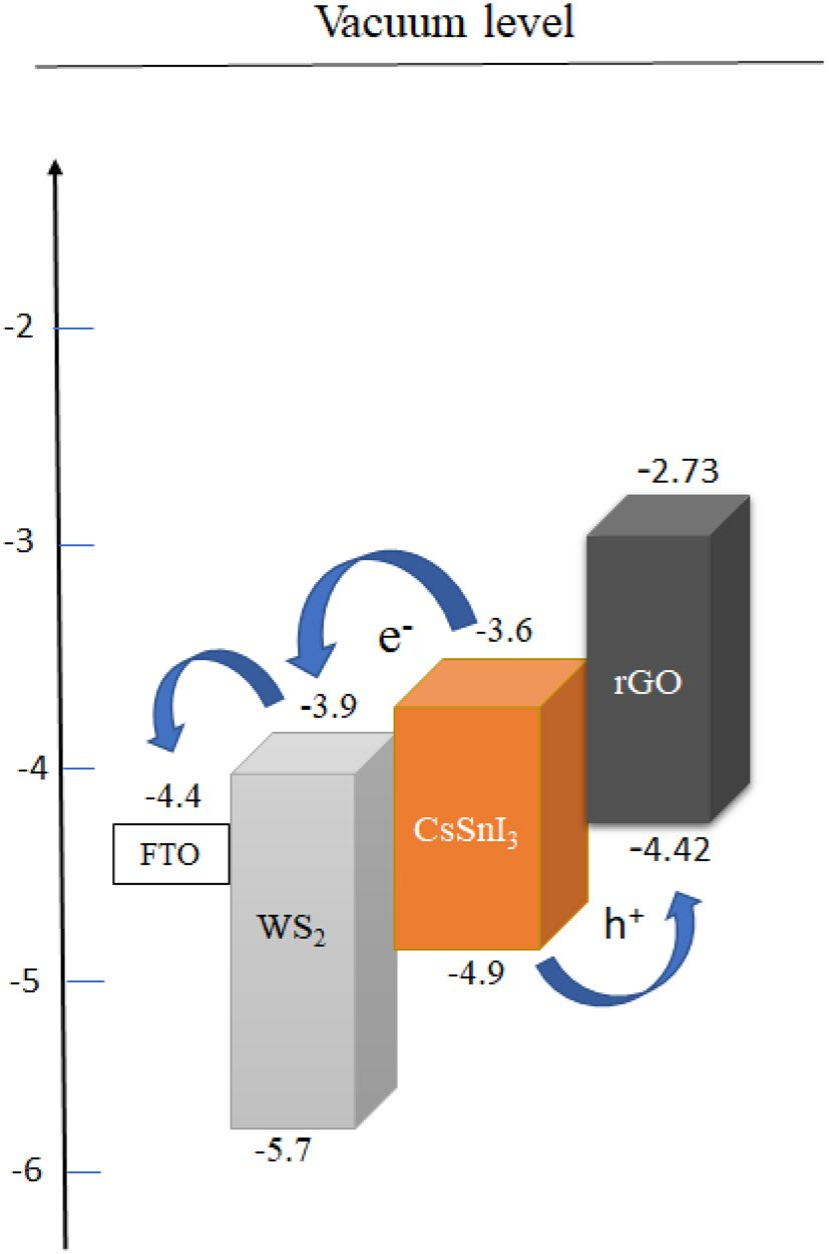
Schematic representation of charge transfer mechanism in lead-free all inorganic PSC structure.

Also, in Fig. 5, we have shown the conduction band minima (CBM) and valance band maxima (VBM) of the perovskite, HTL and ETL layer of the device. It is shown that the energy difference in between the CBM of WS_2_ and absorber layer is smaller than that of the VBM of WS_2_ and absorber layer. As a result, it allows the easy conduction of electron from the conduction band of absorber to FTO through WS_2_ layer and blocks the conduction of holes from VBM of absorber to VBM of WS_2_ layer. Similarly, the difference in energy between the CBM of absorber layer and rGO is larger than that of the VBM of absorber and rGO layer. These differences allow the easy conduction of holes from VBM of absorber to the VBM of rGO and blocks the electron conduction from CBM of absorber to CBM of rGO layer.

### Thickness variation of absorber layer

**Figure 6:**
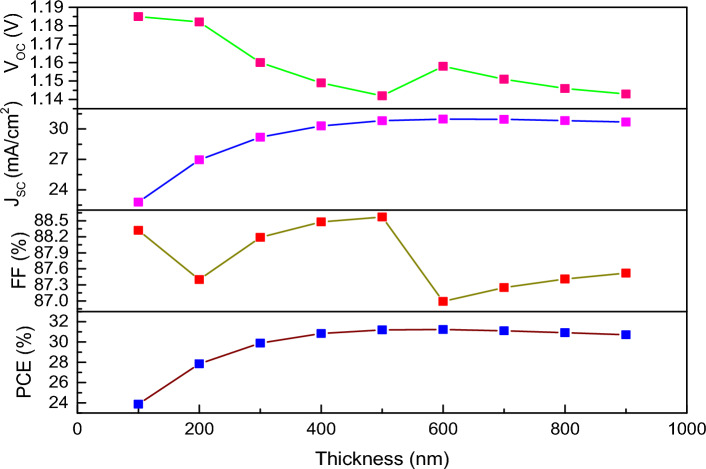
Analysis of photovoltaic parameters with the variation in thickness of active layer for the device (FTO/WS_2_/CsSnI_3_/rGO).

The thickness of absorber layer is the most important factor for variation in photovoltaic parameters (i.e., PCE, FF, J_SC_ and V_OC_) and for photo generated carriers (electron and hole) generated in this layer as incident light is absorbed^70^. From Fig. 6, we can observe the impact of variation in thickness on the device performance of FTO/WS_2_/CsSnI_3_/rGO structured one. It is observed that J_SC_ of the device increases gradually with the increase of thickness from 100 to 500 nm of active layer (optimum value) and then decreases slightly with the increase in thickness. The reason behind the increase in J_SC_ is due to the increase in charge carrier generation by enormous amount of light absorption, but J_SC_ value decreases slightly when the carrier recombination is more dominant with the increase of thickness^62^. PCE also follows the same trend whereas V_OC_ decreases with increase in thickness due to the reduction in diffusion length of charge carriers which results carrier recombination. The V_OC_ reaches its minimum value at 500 nm, so we consider 400 nm as the optimum value instead of 500 nm for our further analysis. Here, FF behaves in a zig-zag manner by variation in thickness of the absorber layer, which may be analysed in future study.

### Variation in temperature

**Figure 7:**
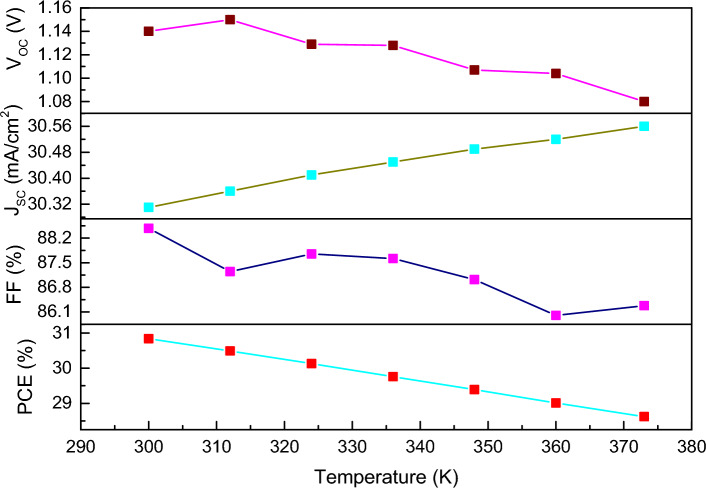
Analysis of photovoltaic parameters with the variation in temperature of FTO/WS_2_/CsSnI_3_/rGO structure.

Environmental factors considerably affect the cell performance including stability and quality of the PSCs. Here we have varied the temperature from 300 K (27 °C) to 373 K (100 °C) by taking other parameters as constant to observe the effect on photovoltaic performance of FTO/WS_2_/CsSnI_3_/rGO structured device. Generally, increase in temperature affects the electron and hole mobility, energy bandgap and absorption coefficient of the different layer in solar cell. Therefore, when temperature increases the carrier recombination increases as a result there is a decrease in PCE value^66^. However, J_SC_ increases with increase in temperature which is associated with the increase in number generation of electrons and holes by decreasing the bandgap^70^. From the Fig. 7, we found the V_OC_ and FF decrease substantially with increase in temperature. This observation can be analysed using the following Eq. (10):

10 $$\frac{d\left({V}_{oc}\right)}{dT}=\frac{{V}_{oc}}{T}-\frac{E_g/q}{T}$$

where the rate of change of V_OC_ is inversely proportional to the temperature. Overall, we found an adverse effect on the PV performance as the temperature increases. Therefore, we consider 300 K as the optimised one for the simulation.

### Defect density variation

It is already explained that the presence of defects in absorber layer is the main cause for electron–hole recombination phenomena, so it behaves as recombination centres or trap sites. This recombination phenomena are associated with the decrement in diffusion length and lifetime of carriers resulting decay in cell performance. This type of recombination is explained by Shockley–Read–Hall effect^71,72^ calculated using the following Eq. (11):

11 $${R}_{SRH}=\frac{np-{n}_{i}^{2}}{{\tau }_{p}\left(n+{N}_{c }{e}^{\frac{{E}_{g}-{E}_{t}}{{K}_{B}T}}\right)+{\tau }_{n}\left(P+{N}_{v}{e}^{\frac{{E}_{t}}{{K}_{B}T}}\right)}$$

where R_SRH_ represents the recombination rate, n and p is the concentration of electron and holes, E_t_ denotes the energy level of trap states, τ_n,p_ is the lifetime of electron and hole. Furthermore, the lifetime of the carriers can be calculated using the following Eq. (12):

12 $${\tau }_{n,p}=\frac{1}{{\sigma }_{n,p}{V}_{th}{N}_{t}}$$

where σ_n,p_ represents the capture cross-sectional area for electrons and holes, N_t_ is the density of trap sites and V_th_ shows the thermal velocity of mobile carriers. The relation between the diffusion length L and lifetime is given using the following Eq. (13)

13 $${L}_{n,p}=\sqrt{\frac{{\mu }_{n,p}{K}_{B}T{ \tau }_{n,p}}{q}}$$

**Figure 8:**
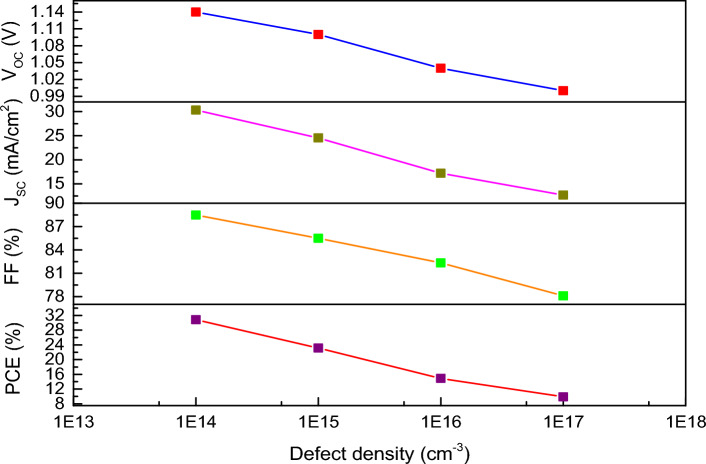
Analysis of photovoltaic parameters with the variation in defect density of perovskite layer for FTO/WS_2_/CsSnI_3_/rGO structure.

Using the above formulae, the following parameters can be calculated such as carrier recombination lifetime and diffusion length with the variation in defect density as listed in Table 5. For which, we have considered V_th_ = 10^7^ cm s^−1^, σ = 10^–15^ cm^2^, K_B_ = 1.381 × 10^–23^ J K^−1^, T = 300 K, q = 1.6 × 10^–19^ C, respectively. From the Table 5, it is clearly identified that the carrier lifetime and the diffusion length decrease gradually with the increase of defect density. Figure 8 exhibits the variation of PV parameters with defect density of the absorber layer from 10^14^ to 10^17^ cm^−3^ in FTO/WS_2_/CsSnI_3_/rGO structured one. It can be noted that the overall cell performance drops with increase of defect density in absorber layer. A highest PCE of 30.84% is achieved at defect density of 10^14^ cm^−3^. Therefore, we take this defect density as optimum value for further analysis.

**Table 5:** Calculation of diffusion length and carrier lifetime with variation in defect density.

N_t_ (cm^−3^)	10^14^	10^15^	10^16^	10^17^
τ_n,p_ (s)	10^–6^	10^–7^	10^–8^	10^–9^
L (µm)	0.90	0.28	0.09	0.028

### Doping density variation

**Figure 9:**
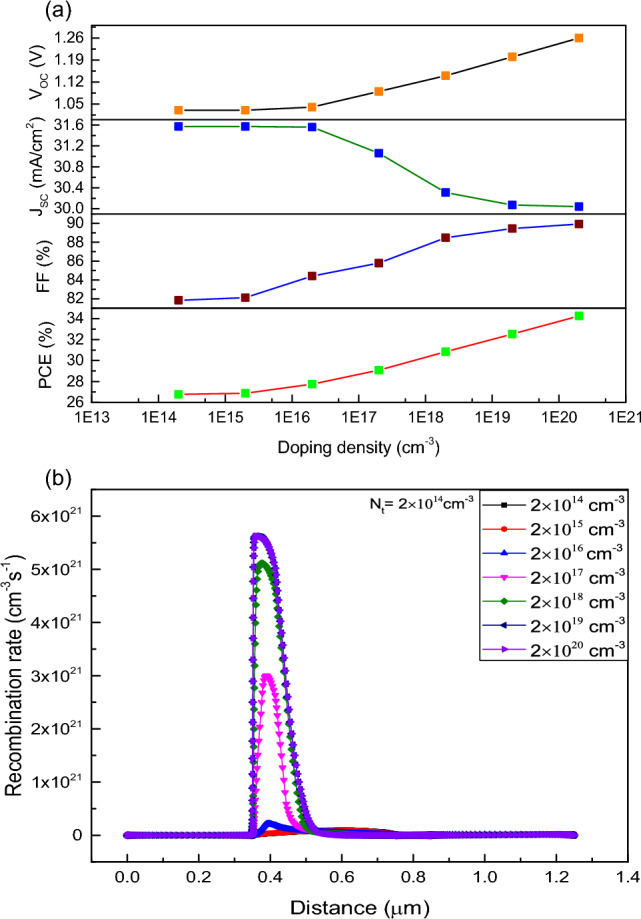
(**a**) Analysis of photovoltaic parameters with the variation in doping density of absorber layer for FTO/WS_2_/CsSnI_3_/rGO, (**b**) variation of recombination rate with depth from surface for different doping density with a defect density of 10^14^ cm^−3^ in the absorber layer.

It can be observed from the Fig. 9a that, increase in doping density will enhance the photovoltaic performance because of the increase in electric potential at perovskite surface. To find the optimum value for dopant concentration, doping density of absorber layer is varied from 2 × 10^14^ to 2 × 10^20^ cm^−3^ for FTO/WS_2_/CsSnI_3_/rGO device structure and it is inferred that, all parameters like FF, PCE, V_OC_ of the device gradually increases except J_SC_. The J_SC_ remains constant up to doping density of 2 × 10^16^ cm ^−3^, then it decreases gradually. Furthermore, the J_SC_ remains again constant from 2 × 10^19^ to 2 × 10^20^ cm^−3^ of doping density. This behaviour of J_SC_ is due to the decrease in diffusion length of minority charge carriers at higher acceptor doping concentration^73^. Because of the opposite trend in variation of J_SC_, we consider 2 × 10^18^ cm^−3^ as the absorber doping density in our further calculation. However, increase in doping concentration increases V_OC_ of the cell because of decrement in reverse saturation current by Eq. (14)^70^.

14 $${V}_{OC }=\frac{nKT}{q}\mathrm{log}\left(\frac{{J}_{SC}}{{J}_{0}}+1\right)$$

Here, J_0_ denotes reverse saturation current, J_SC_ is the short circuit current, n is the ideality factor, T is the temperature and K is the Boltzmann constant, respectively. Also, FF and PCE increase due to increase in number of charge carriers. From the above analysis it can be concluded that using the optimum doping concertation we can get an improved V_OC_ and FF simultaneously with increased PCE value. At a particular defect density of 10^14^ cm^−3^, we analysed the recombination phenomenon with the variation of doping concentration from 2 × 10^14^ to 2 × 10^20^ cm^−3^ for the absorber layer of PSC. Here, Fig. 9b shows that at constant defect density if we increase the doping concentration, the recombination rate increases, and thus, it shows reduction in the diffusion length of mobile charge carriers.

### Variation of electrode

Here, we have simulated the PSC structure (FTO/WS_2_/CsSnI_3_/rGO) using different back contact electrodes i.e., Silver (Ag), Copper (Cu), Gold (Au), Nickel (Ni), Palladium (Pd), Platinum (Pt) and Selenium (Se) having different work function^46,73^. The work function of these materials and the PCE obtained from simulation using these as electrodes in the device are listed in Table 6. From where, we observed that with the increase in work function, the PCE of device increases. The back contact of the solar cell forms a junction with the HTL (rGO). If work function of the electrode is less than the HTL, then it prohibits the flow of holes from HTL due to the Schottky barrier formation at the junction^50,74^. To avoid this effect, the selection of proper back electrode having higher work function value is very important. Also, we obtained similar PCE (31%) value for Pt, Pd and Se electrodes. But, due to the toxic nature of Se and Pd, it is better to select Pt as a suitable back contact electrode for the present study. A comparative study of PV parameters with respect to other works as reported elsewhere are listed in Table 7. Furthermore, using Pt electrode we have achieved a PCE of 20.29% for FTO/WS_2_/CsPbI_3_/rGO/Pt structured device. The selected electrode (Pt) forms an ohmic contact instead of Schottky contact at the rGO-Pt interface and this can be represented by the given Eq. (15)^46^.

15 $${\phi }_{B}=\frac{{E}_{g}}{q}+ \chi -{\phi }_{m}$$

where ${\phi }_{B}$ denotes the surface potential barrier at the anode-rGO interface. ${E}_{g}$ is the band gap of rGO, χ is the electron affinity of rGO and ${\phi }_{m}$ is the electrode work function. Therefore, decrease in value of work function, the surface potential energy barrier increases, the PCE decreases obtained by the above Eq. (15).

**Table 6:** Work function and corresponding PCE to several metal electrodes.

Electrode	Work function (eV)	PCE (%)
Ag	4.74	30.58
Cu	5.0	30.82
Au	5.1	30.82
Ni	5.5	30.97
Pd	5.6	31.0
Pt	5.7	31.0
Se	5.9	31.0

**Table 7:** Comparative study of simulation based CsSnI_3_ perovskite solar cell.

Device structure	V_OC_ (V)	J_SC_ (mA cm^−2^)	FF (%)	PCE (%)	References
Ga-ITO/NiO_X_/CsSnI_3_/PCBM/Ag	1.19	17.29	55.27	11.41	^75^
TCO/PCBM/CsSnI_3_/CuI/Au	0.89	34.78	85.14	26.52	^76^
FTO/TiO_2_/CsSnI_3_/spiro-OMeTAD/Au	1.06	16.67	88.40	15.70	^47^
FTO/TiO_2_/CsSnI_3_/Spiro-OMeTAD/Au	1.90	28.85	88.65	28.09	^30^
ITO/TiO_2_/CsSnI_3_/CuS	0.99	33.50	88.60	28.76	^31^
ITO/PCBM/CsSnI_3_/CFTS/Se	0.872	33.99	83.46	24.73	^29^
FTO/TiO_2_/CsSnI_3_/P_3_HT	0.97	34.70	78.21	26.40	^32^
FTO/N-TiO_2_/CsSnI_3_/Spiro-OMeTAD/Au	0.98	34.69	79.45	27.03	^77^
ITO/PCBM/CsSnI_3_/CuI/Au	0.91	14.24	78.11	10.10	^78^
FTO/TiO_2_/CsSnI_3_/Cu_2_ZnSnSe_4_/Au	0.83	34.38	75.43	21.63	^79^
FTO/TiO_2_/CsSnI_3_/CuSCN/Au	1.01	29.82	84.15	25.42	^80^
FTO/WS_2_/CsSnI_3_/rGO/Pt	1.15	30.47	88.48	31.00	This work

### Variation of R_S_ and R_Sh_

**Figure 10:**
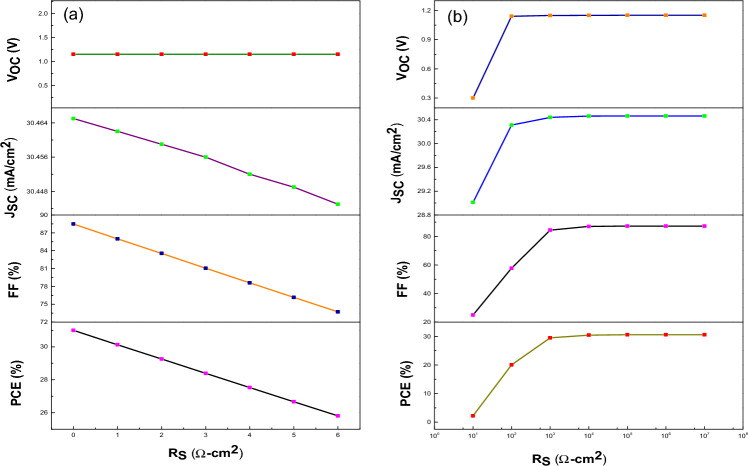
Analysis of photovoltaic parameters with the variation of (**a**) series resistance (R_S_) and (**b**) shunt resistance (R_Sh_) in the device (FTO/WS_2_/CsSnI_3_/rGO/Pt).

It is already proven that the variation in series resistance (R_S_) and shunt resistance (R_Sh_) has a significant impact on the PV parameter especially on FF and J_SC_. The R_S_ is attributed to ohmic resistance present in between the junction of HTL and electrode whereas R_Sh_ is contributed to defects and trap assisted recombination mechanism carried out inside the device. In this work, we have varied the R_S_ from 0 to 6 Ω-cm^2^ and the R_Sh_ varied from 10 to 10^7^ Ω-cm^2^ with constant R_S_ of 0.5 Ω-cm^2^ (due to the presence of some amount of sheet resistance in the device). Figure 10a shows the effect of R_S_ variation on PV performance of FTO/WS_2_/CsSnI_3_/rGO/Pt structured cell. The FF, PCE and J_SC_ of device decrease gradually with increase in R_S_. The variation in R_S_ does not affect the V_OC_ as it behaves constant during the entire process. The R_S_ is associated with device current density followed by Eq. (16):

16 $$J={J}_{L}-{J}_{0}exp\left[\frac{q\left(V+I{R}_{S}\right)}{nKT}\right]$$

whereas the relation between the R_S_ and FF is given by Eq. (17)^73^.

17 $${FF}_{S}={FF}_{0}\left(1-{R}_{S}\right)$$

where FF_0_ is the FF in absence of R_S_ and R_Sh_ and FF_S_ is the FF in presence of series resistance. Figure 10b exhibits the effect of variation in R_Sh_ on the PV parameters of FTO/WS_2_/CsSnI_3_/rGO/Pt structured device. All the parameters increase up to a certain level for increase in R_Sh_, and after that it provides a stable cell performance with further increase in R_Sh_ value. The FF and output current variation in presence of R_Sh_ are given by the following Eqs. (18) and (19)^73^.

18 $$J={J}_{L}-{J}_{0}exp\left[\frac{q\left(V+I{R}_{S}\right)}{nKT}\right]-\frac{V+I{R}_{S}}{{R}_{Sh}}$$

19 $${FF}_{Sh}={FF}_{0}\left(1-\frac{1}{{R}_{Sh}}\right)$$

where FF_Sh_ represents the FF in presence of shunt resistance. Figure S2a–d (contour plots) show the combinational effect of series and shunt resistance on PV parameters. From where we observed that the region having maximum value of R_Sh_ and minimum value of R_S_ shows enhancement in photovoltaic performances. From the Fig. 10a, we obtained a highest PCE of 31% for a R_S_ of 0 Ω-cm^2^. It is experimentally verified that R_S_ never become zero due to the sheet resistance of substrates whereas all the photovoltaic parameters are constant from R_Sh_ of 10^5^ Ω-cm^2^ as shown in Fig. 10b. Therefore, we consider a R_S_ of 0.5 Ω-cm^2^ and R_Sh_ of 10^5^ Ω-cm^2^ for further analysis. It is found that for an ideal solar cell the FF must be 1. But due to presence of some unavoidable losses arisen from the internal resistances (i.e., R_S_ and R_Sh_) and recombination of carriers (Auger, SRH, Band-to-Band), it will be less than unity (real model). The upper limit for the fill factor (FF) could be obtained 89% as reported elsewhere^81^. In the present study, we obtained FF of 88.47% for the rGO as a HTL and WS_2_ as a ETL in lead-free device which approaches the upper limit as described above.

### Electrical parameter analysis

**Figure 11:**
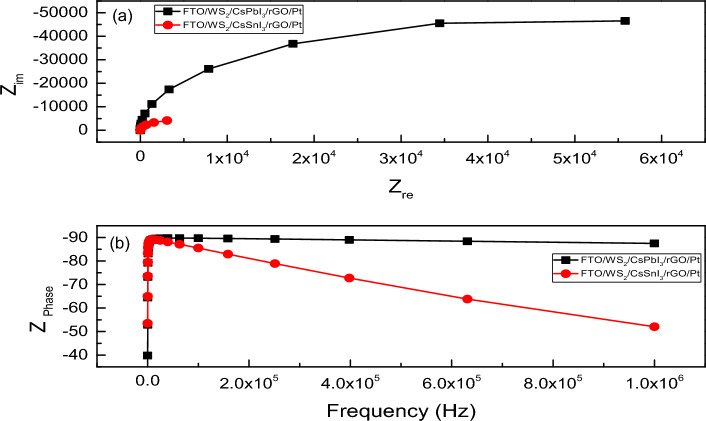
(**a**) Nyquist plot, (**b**) Bode plot of FTO/WS_2_/CsPbI_3_/rGO/Pt and FTO/WS_2_/CsSnI_3_/rGO/Pt structures.

**Figure 12:**
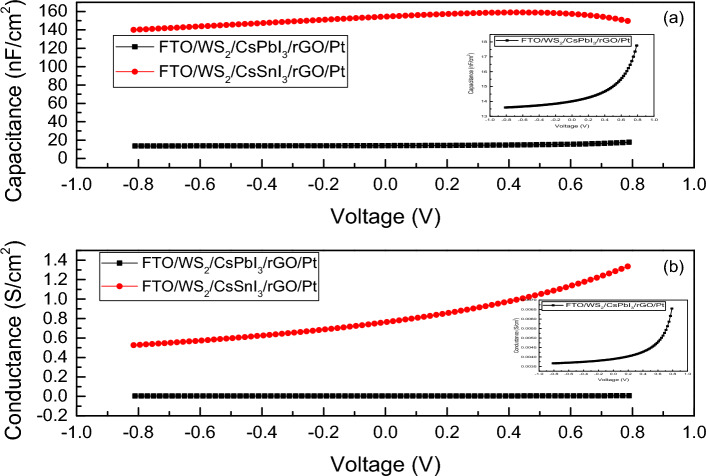
(**a**) Capacitance vs. voltage plot, (**b**) conductance vs. voltage plot for FTO/WS_2_/CsPbI_3_/rGO/Pt and FTO/WS_2_/CsSnI_3_/rGO/Pt with close view of both the graphs for FTO/WS_2_/CsPbI_3_/rGO/Pt (inset).

Electrical impedance spectroscopy (EIS) is a technique used to characterize different phenomena (electronic and ionic process) running inside the PSCs i.e., recombination process, charge accumulation process at the interfaces, carrier transport mechanism, especially the transport rate, variation of capacitance and conductance, etc.^82^. Basically, the complex impedance plots i.e., Nyquist Plot (Z′ vs. Z″) and Bode plot (Frequency vs. Z_Phase_) describe the carrier transport mechanism with a wide range of frequency associated with different resonance time. The Nyquist plot provides the effect of RC component associated in circuit whereas Bode plot indicates the achievable peak frequency. The size or width of arc in Nyquist plot provides the nature of charge transfer and the resistance associated with it^83^. For electrical analysis, we consider R_S_ and R_Sh_ to be 0.5 Ω-cm^2^ and 10^5^ Ω-cm^2^, respectively and a comparison of electrical performance is conducted among the Pb-based and Pb-free structures with rGO as HTL and WS_2_ as ETL. Figure 11a shows the semi-circular arc of Nyquist plot formed due to the presence of parallel RC component in the circuit for FTO/WS_2_/CsSnI_3_/rGO/Pt and FTO/WS_2_/CsPbI_3_/rGO/Pt structured devices. Figure 11b represents the frequency variation of Bode plot and gives a peak frequency of 0.015 MHz and 0.01 MHz for device structure of FTO/WS_2_/CsPbI_3_/rGO/Pt and FTO/WS_2_/CsSnI_3_/rGO/Pt, respectively. Accordingly, the associated resonance time are calculated to be 63 µs and 100 µs, respectively using the following Eq. (20):

20 $$T=\frac{1}{f}$$

where T denotes the resonance time and *f* denotes the associated frequency. Figure 12a shows the highest capacitance of 158.93 nF cm^−2^ for FTO/WS_2_/CsSnI_3_/rGO/Pt based one, meanwhile the capacitance of 17.76 nF cm^−2^ is detected for FTO/WS_2_/CsPbI_3_/rGO/Pt based device. It is reported that increased value of capacitance corresponds to the absorption of lower wavelength photons^50^. Figure 12b represents the conductance vs. voltage relationship, where FTO/WS_2_/CsSnI_3_/rGO/Pt structure has the highest value of conductance i.e., 1.33 S cm^−2^ at 0.78 V. The conductance of 0.0065 S cm^−2^ is obtained for FTO/WS_2_/CsPbI_3_/rGO/Pt structured one. This result speaks about the high radiation value induced at that voltage^84^. Figure S3a,b represent the capacitance vs. frequency and conductance vs. frequency relationship provided the high value of capacitive response in low frequency range related to ionic movement and it can be used to analyse value of dielectric constant^85^. Figure S3c,d provide the complex capacitance data as it is related with frequency obeys the following Eqs. (21) and (22):

21 $$C\left(\omega \right)=\frac{1}{i\omega Z(\omega )}$$

22 $$\omega =2\pi f$$

where ω represents the angular frequency.

### Variation of illumination intensity

We already obtained the optimal value for series (R_S_) and shunt resistance (R_Sh_) from the above analysis. Now we vary the series and shunt resistance with incident light intensity to examine its effect on PV parameters of FTO/WS_2_/CsSnI_3_/rGO/Pt structured PSCs or on the overall cell performance. The value of R_S_ from 0 to 2 Ω-cm^2^ and R_Sh_ from 10^2^ to 10^5^ Ω-cm^2^ are varied in this study.

**Figure 13:**
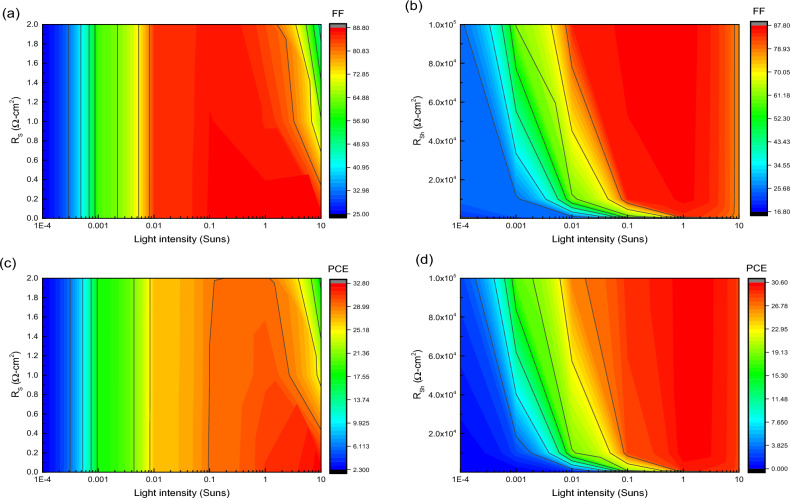
Contour plot of (**a**) R_S_, and FF vs. light intensity, (**b**) R_Sh_, and FF vs. light intensity, (**c**) R_S_ and PCE vs. light intensity, (**d**) R_Sh_ and PCE vs. light intensity for FTO/WS_2_/CsSnI_3_/rGO/Pt structured device.

From the Fig. 13a, it is obvious that, the increase in R_S_ reduces FF of the device. The effect of R_S_ on FF becomes less prominent with the decrease in light intensity, and it appears more significant at high light intensity region. From the Fig. 13b, it must be demonstrated that the increment in R_Sh_ enhances FF of the device. The optimum FF is measured in high light intensity region with high R_Sh_ value, whereas the low value of R_Sh_ is visible only at low light intensity region. The PCE of device also follows the same trend as FF with the variation of Rs and R_Sh_, which are shown in Fig. 13c,d, respectively.

#### Illumination intensity and Ideality factor

The light intensity study of J–V curves not only says about the photovoltaic parameters but also provides the insight into different type of recombination occurs at the bulk or interfaces of the junction layers. Here, the ideality factor comes into consideration, which provides knowledge about the dominant recombination mechanism at a particular region in different layers of the device^86^. The variation of ideality factor and reverse saturation current with different light intensity gives an idea about electrical conduction at the junction. It is reported that, ideality factor can be estimated from the slope of Log(J + J_SC_) vs. Voltage plot for illumination condition and Log(J) vs. Voltage plot for dark condition without consideration of series and shunt resistance in the circuit. In standard condition of small R_S_ and large R_Sh_ value, the ideality factor can be determined from the slope q/nKT of straight line obtained from Log(J + J_SC_) vs. V′ plot. Where V′ is taken as V + J_SC_R_S_^87^.

**Figure 14:**
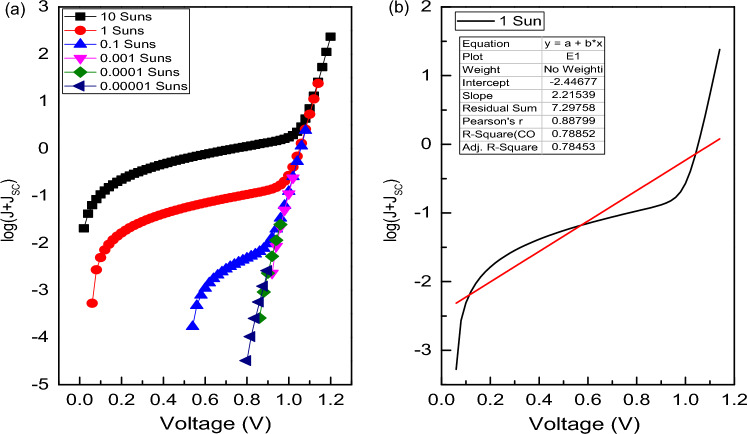
(**a**) J–V curve for different intensity, (**b**) Log(J + J_SC_) vs. V plot for determination of ideality factor under 1 Sun illumination in absence of R_S_ and R_Sh_ for FTO/WS_2_/CsSnI_3_/rGO/Pt structured device.

Figure 14a exhibits the J–V variation for different light intensity from 10^–4^ to 10 Suns illuminations in absence of R_S_ and R_Sh_ for FTO/WS_2_/CsSnI_3_/rGO/Pt structure. The ideality factor of 2.21 KT/q for 1 Sun illumination can be extracted from the graph as shown in Fig. 14b. From the Table 8, it is observed that ideality factor increases up to 0.01 Suns intensity then, it gradually decreases with the decrease of light intensity. It is generally found that the low light intensity region is affected by the bulk recombination, which further gives rise to the increment in ideality factor with decrease in light intensity. However, the high light intensity region is affected by interface recombination.

**Table 8:** Ideality factor variation with light intensity in absence of R_S_ and R_Sh_.

Illumination intensity (Suns)	Ideality factor (KT/q)
10	1.86
1	2.21
0.1	5.74
0.01	19.70
0.001	19.35
0.0001	18.69

**Figure 15:**
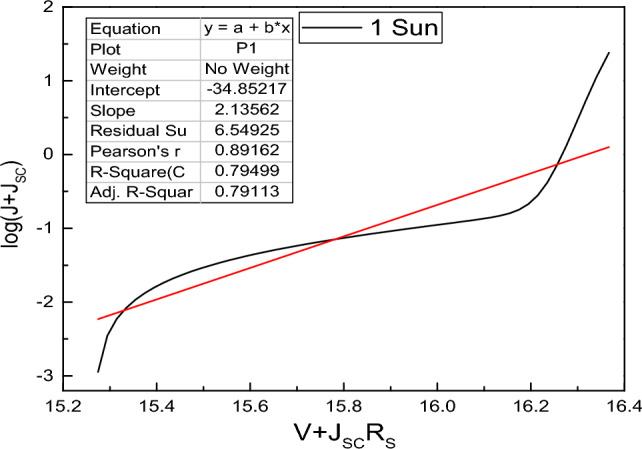
Determination of ideality factor in presence of R_S_ and R_Sh_ for FTO/WS_2_/CsSnI_3_/rGO/Pt structured device.

From Fig. 15, we obtained the ideality factor of 2.13 KT/q in presence of R_S_ = 0.5 Ω-cm^2^ and R_Sh_ = 10^5^ Ω-cm^2^ for one sun illumination of FTO/WS_2_/CsSnI_3_/rGO/Pt structure. All the ideality factor associated with the light intensities varying from 0.0001 to 10 Suns are listed in Table 9. We observe a zig-zag pattern of ideality factor variation with the decrement in light intensity. If we see the light intensity variation from 10 Suns to 1 Sun, ideality factor decreases signifying the enhancement of interface recombination. Then ideality factor gradually increases up to 0.01 Suns. When light intensity variation from 0.01 to 0.0001 Suns, it shows a zig-zag pattern of ideality factor. It is reported that the value of ideality factor ranges from 1 to 1.5 KT/q provided the domination of interface recombination^86^ and domination of SRH recombination is found for ideality factor of 1–2 KT/q^88^. In this study, the Auger recombination is neglected as it shows its effectiveness at very high light intensity (i.e., for high carrier concentration region).

**Table 9:** Ideality factor variation with light intensity in presence of R_S_ and R_Sh_.

Illumination intensity (Suns)	Ideality factor (KT/q) with Pt electrode
10	2.3
1	2.13
0.10	3.15
0.01	3.70
0.001	1.48
0.0001	1.56

## Conclusion

A thorough study of regular n–i–p structured PSCs is performed by varying the HTLs (both the organic and inorganic) and then ETLs for the device. After the variation of HTLs and ETLs, highest efficiency of 30.84% is achieved from FTO/WS_2_/CsSnI_3_/rGO structured device. Then the optimised structure is simulated to examine the photovoltaic performance by varying the device temperature, thickness, defect density and doping density of absorber layer, back contact electrodes (BCE), series resistance (R_S_) and shunt resistance (R_Sh_), etc. By taking optimum thickness of 400 nm, doping density of 2 × 10^18^ cm^−3^, defect density of 10^14^ cm^−3^ and Pt as BCE, we have achieved a maximum efficiency of 31% for FTO/WS_2_/CsSnI_3_/rGO/Pt (lead-free) structured device whereas the PCE obtained for FTO/WS_2_/CsPbI_3_/rGO/Pt (lead-based) is 20.29% only. A comparative study is performed through Nyquist plot, Bode plot, and capacitance, conductance value of FTO/WS_2_/CsSnI_3_/rGO/Pt as well as FTO/WS_2_/CsPbI_3_/rGO/Pt structured devices by taking into consideration R_S_ of 0.5 Ω-cm^2^ and R_Sh_ of 10^5^ Ω-cm^2^, respectively. The CsSnI_3_ based device exhibited a capacitance and conductance of 158.93 nF cm^−2^ and 1.33 S cm^−2^, respectively whereas CsPbI_3_ based one exhibited a capacitance and conductance of 17.76 nF cm^−2^ and 0.0065 S cm^−2^, respectively at 0.78 V. Furthermore, the effect of light intensity variation on the photovoltaic performance is also optimised. Mostly FF is affected by the simultaneous variation of light intensity and ohmic resistances. Also, ideality factor analysis is carried out to estimate the dominant recombination mechanism in the interfaces between the layers of device. Therefore, this analysis will provide an insight on the fabrication process of lead-free all inorganic perovskite solar cell for researchers.

### Supplementary Information

Supplementary Information.

## Data Availability

All data generated or analysed during this study are included in this published article [and its supplementary information files].
