# Author Correction: All-inorganic perovskite photovoltaics for power conversion efficiency of 31%

**DOI:** 10.1038/s41598-023-44173-9

**Published:** 2023-10-10

**Authors:** Lipsa Rani Karna, Rohitash Upadhyay, Avijit Ghosh

**Affiliations:** Department of Physics, Central University of Jharkhand, Ranchi, 835222 India

Correction to: *Scientific Reports* 10.1038/s41598-023-42447-w, published online 14 September 2023

The original version of this Article contained errors in Table 7. The PCE (%) for “ITO/TiO_2_/CsSnI_3_/CuS” was incorrectly given as “28.76”. Moreover, the device structure “ITO/PCBM/CsSnI_3_/CFTS/Se” was incorrectly given as “ITO/PCBM/CsSnI_3_/CuSCN/Se”.

The incorrect and correct device structures and PCEs appear below.

Incorrect:

**Table:** 

Device structure	V_OC_ (V)	J_SC_ (mA/cm^-2^)	FF (%)	PCE (%)	Reference
ITO/TiO_2_/CsSnI_3_/CuS	0.99	33.50	88.60	28.78	^31^
ITO/PCBM/CsSnI_3_/CuSCN/Se	0.872	33.99	83.46	24.73	^29^

Correct:

**Table:** 

Device structure	V_OC_ (V)	J_SC_ (mA/cm^-2^)	FF (%)	PCE (%)	Reference
ITO/TiO_2_/CsSnI_3_/CuS	0.99	33.50	88.60	28.76	^31^
ITO/PCBM/CsSnI_3_/CFTS/Se	0.872	33.99	83.46	24.73	^29^

The original Article has been corrected.

