# Supplementary material for: All-inorganic perovskite photovoltaics for power conversion efficiency of 31%
Source: Sci Rep. 2023 Sep 14;13:15212. doi: 10.1038/s41598-023-42447-w (PMC10502065; doi:10.1038/s41598-023-42447-w)
Supplement: Supplementary file 1 — Supplementary Information. [file 41598_2023_42447_MOESM1_ESM.docx]

**All-inorganic Perovskite Photovoltaics for Power Conversion Efficiency of 31 %**

Lipsa Rani Karna, Rohitash Upadhyay, and Avijit Ghosh*

Department of Physics, Central University of Jharkhand, Ranchi-835222, India

**Supplementary Information**

**Fig. S1 (a) J-V curves and (b) External Quantum Efficiency variation curves** **for** **CsSnI_3_ and CsPbI_3_ based devices with rGO as HTL and WS_2_ as ETL**

**Fig. S2 (a), (b), (c), (d) Analysis of photovoltaic parameters with the concurrent variation of R_S_ and R_Sh_ for FTO/WS_2_/CsSnI_3_/rGO/Pt structured device**

**Fig. S3 (a), (c) Capacitance vs. Frequency plot and (b), (d) Conductance vs. Frequency plot of** **FTO/WS_2_/CsPbI_3_/rGO/Pt, and FTO/WS_2_/CsSnI_3_/rGO/Pt with close up view of every plot for FTO/WS_2_/CsPbI_3_/rGO/Pt (Inset)**
